# The Protective Effects of Isoliquiritigenin and Glycyrrhetinic Acid against Triptolide-Induced Oxidative Stress in HepG2 Cells Involve Nrf2 Activation

**DOI:** 10.1155/2016/8912184

**Published:** 2016-01-21

**Authors:** Ling-Juan Cao, Huan-De Li, Miao Yan, Zhi-Hua Li, Hui Gong, Pei Jiang, Yang Deng, Ping-Fei Fang, Bi-Kui Zhang

**Affiliations:** ^1^Department of Pharmacy, The Second Xiangya Hospital, Central South University, Changsha 410011, China; ^2^Institute of Clinical Pharmacy, Central South University, Changsha 410011, China; ^3^School of Pharmaceutical Sciences, Central South University, Changsha 410013, China

## Abstract

Triptolide (TP), an active ingredient of* Tripterygium wilfordii* Hook f., possesses a wide range of biological activities. Oxidative stress likely plays a role in TP-induced hepatotoxicity. Isoliquiritigenin (ISL) and glycyrrhetinic acid (GA) are potent hepatoprotection agents. The aim of the present study was to investigate whether Nrf2 pathway is associated with the protective effects of ISL and GA against TP-induced oxidative stress or not. HepG2 cells were treated with TP (50 nM) for 24 h after pretreatment with ISL and GA (5, 10, and 20 *μ*M) for 12 h and 24 h, respectively. The results demonstrated that TP treatment significantly increased ROS levels and decreased GSH levels. Both ISL and GA pretreatment decreased ROS and meanwhile enhanced intracellular GSH content. Additionally, TP treatment obviously decreased the protein expression of Nrf2 and its target genes including HO-1 and MRP2 except NQO1. Moreover, both ISL and GA displayed activities as inducers of Nrf2 and increased the expression of HO-1, NQO1, and MRP2. Taken together the current data confirmed that ISL and GA could activate the Nrf2 antioxidant response in HepG2 cells, increasing the expression of its target genes which may be partly associated with their protective effects in TP-induced oxidative stress.

## 1. Introduction

Triptolide (TP), a major active ingredient extracted from the widely used Chinese herb* Tripterygium wilfordii* Hook f. (TWHF), has been demonstrated to possess various biological activities including anti-inflammation, immune modulation, and antiproliferative activity [1]. However, the clinical application of TP is limited by its narrow therapeutic window and severe toxicities involving hepatotoxicity, nephrotoxicity, and reproductive toxicity [1]. Among these, hepatotoxicity has drawn great attention [2]. A possible mechanism of TP-induced hepatotoxicity was related to oxidative stress damage caused by reactive oxygen species (ROS) [2].

The nuclear factor erythroid 2-related factor 2 (Nrf2) is an emerging regulator of cellular resistance to substances causing oxidative stress [3]. Under physiological condition, Nrf2 is present in the cytoplasm binding to the Kelch-like ECH-associated protein 1 (Keap1). Under stress condition, Nrf2 dissociates from Keap1 and translocates into the nuclear where it binds to antioxidant responsive element (ARE), leading to the expression of its target genes [4]. These include many cytoprotective proteins and drug efflux transporters, such as heme oxygenase 1 (HO-1), NAD (P) H: quinone oxidoreductase (NQO1), glutamate cysteine ligase (GCL), and multidrug resistance-associated protein 2 (MRP2) [5, 6]. Nrf2 pathway is considered as an important endogenous antioxidant signaling pathway and increasing number of studies suggested the potential role of Nrf2 as a therapeutic target to prevent liver injury caused by oxidative stress [7, 8].

**Figure 1:**
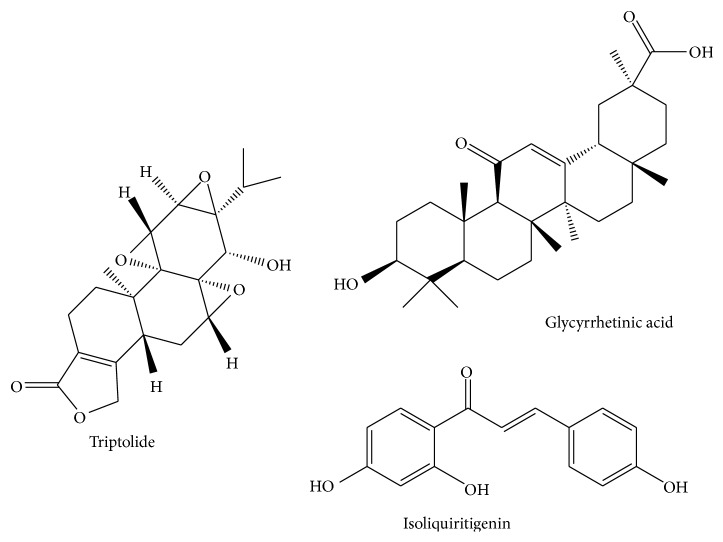
The structures of TP, GA, and ISL.

Licorice (*Glycyrrhiza uralensis*), a common used Traditional Chinese Medicine (TCM), contains various bioactive compounds including triterpene saponins (mainly glycyrrhizin and its bioactive metabolite glycyrrhetinic acid (GA)), flavonoids (such as isoliquiritigenin (ISL)), coumarin, alkaloids, polysaccharides, and amino acids. ISL and GA are two main biologically active components for their useful pharmacological properties such as antioxidation, anti-inflammation, hepatoprotection, and antiviral activity [9]. The structures of ISL and GA are shown in Figure 1.

Licorice is often in combination with TWHF clinically for its toxicity attenuation and efficacy potentiation effect on the treatment of rheumatoid arthritis [1, 10]. Moreover, glycyrrhizin acid has a synergistic effect with TP, thus making TP effective at a lower dosage which may reduce the toxicity of TP since this toxicity is dose-dependent [11]. However, details of the hepatoprotection mechanisms remain to be elucidated. In our previous study, we demonstrated that licorice may intervene in the Nrf2 pathway to induce its target genes, which indicate a novel mechanism for the use of licorice to lower drug toxicity [12]. Based on this observation, our objective was to investigate the regulation effect of ISL and GA on Nrf2 pathway and the protective effects of ISL and GA on TP-induced oxidative stress in human hepatocarcinoma HepG2 cells.

## 2. Materials and Methods

### 2.1. Chemicals and Reagents

ISL (purity > 99%), GA (purity > 99%), and TP (purity ≥ 98%) were purchased from On-Road Biotechnology Co., Ltd. (Changsha, China).* tert*-butylhydroquinone (tBHQ), dimethyl sulfoxide (DMSO), and methyl thiazolyl tetrazolium (MTT) were purchased from Sigma-Aldrich (St. Louis, MO, USA). Glutathione (GSH) Detection Kit was purchased from Nanjing Jiancheng Bioengineering Institute (Nanjing, China). Reactive Oxygen Species Assay Kit was purchased from Beyotime Institute of Biotechnology (Shanghai, China). Nrf2 antibody was purchased from Santa Cruz Biotechnology (Santa Cruz, CA, USA). HO-1, NQO1, and MRP2 antibodies were purchased from Abcam Biotechnology Co. (Milton, Cambridge, UK). Other chemicals were of analytical grade from commercial suppliers.

### 2.2. Cell Culture

Human hepatocarcinoma cell line HepG2 obtained from Xiangya Cell Bank (Changsha, China) was cultured in Dulbecco Modified Eagle's Medium (Hyclone, Logan, USA) supplemented with 10% (v/v) fetal bovine serum (Sijiqing, Hangzhou, China) in a 37°C incubator with 5% CO_2_. ISL, GA, tBHQ, and TP were dissolved in DMSO and stock solutions were stored at −20°C. Drugs were freshly diluted to the indicated concentrations with culture medium before use. DMSO concentration in experimental conditions never exceeded 0.1% (v/v).

### 2.3. MTT Assay

Cell viability was determined by MTT assay. Briefly, cells were counted and plated in a 24-well tissue culture plate and the seeding densities were 2 × 10^4^ cells/well. After 24 h of growth, cells were preincubated with ISL, GA (0, 5, 10, 20, 40, 60, 80, and 100 *μ*M) and TP (0, 10, 20, 40, 50, 60, 80, and 100 nM), respectively, for desired time. Then MTT (5 mg/mL) was added to each well (300 *μ*L) and incubated for 3–5 h, after which the MTT was removed and DMSO (300 *μ*L) was added to each well. After shaking for 10 min, each sample was transferred to a 96-well microtiter plate and the absorbance was recorded at 490 nm.

### 2.4. Measurement of Intracellular ROS

The fluorescent probe DCFH-DA was used to determine the intracellular accumulation of ROS. HepG2 cells were seeded in 96-well black plates at a density of 5 × 10^3^ cells/well and then exposed to various concentrations of ISL, GA for 12 h and 24 h, respectively. After that the complete medium was removed and replaced with medium containing TP (50 nM) for 24 h. Then, the cells were washed with serum-free medium and incubated with DCFH-DA (10 *μ*M) for 20 min at 37°C. The fluorescent intensity was measured at an excitation wavelength of 488 nm and emission wavelength of 525 nm.

### 2.5. GSH Detection

For the measurement of GSH content, HepG2 cells were seeded in 60 cm^2^ dishes with 6 × 10^5^ cells/well. Cells were exposed to drugs as described above, after which cells were harvested and lysed by ultrasonication. Following centrifugation at 3500 ×g for 10 min at 4°C, the supernatant was maintained on ice until assayed by GSH detection Kit according to the manufacturer's instructions.

### 2.6. Preparation of Cell Lysates and Western Blotting

Cells were lysed with RIPA buffer (CW biotech, Beijing, China) and equivalent amounts of protein were separated by 10% SDS-PAGE and transferred to PVDF membranes. After being blocked in 5% nonfat milk in TBST for 1 h at room temperature, the membranes were incubated with the primary antibodies at 4°C overnight. Subsequently, the immunoblots were then incubated with a secondary antibody at room temperature. The membranes were developed using an electrochemiluminescence (ECL) kit (Advansta, USA) according to the manufacturer's protocol.

### 2.7. Statistics

Results from the experiments were reported as mean ± standard deviation (SD) and conducted with SPSS 19.0. All data were analyzed by one-way ANOVA, followed by Tukey's test. Statistical significance was accepted at a *p* value less than 0.05.

## 3. Results

### 3.1. Cytotoxicity Effects of TP, ISL, and GA

**Figure 2:**
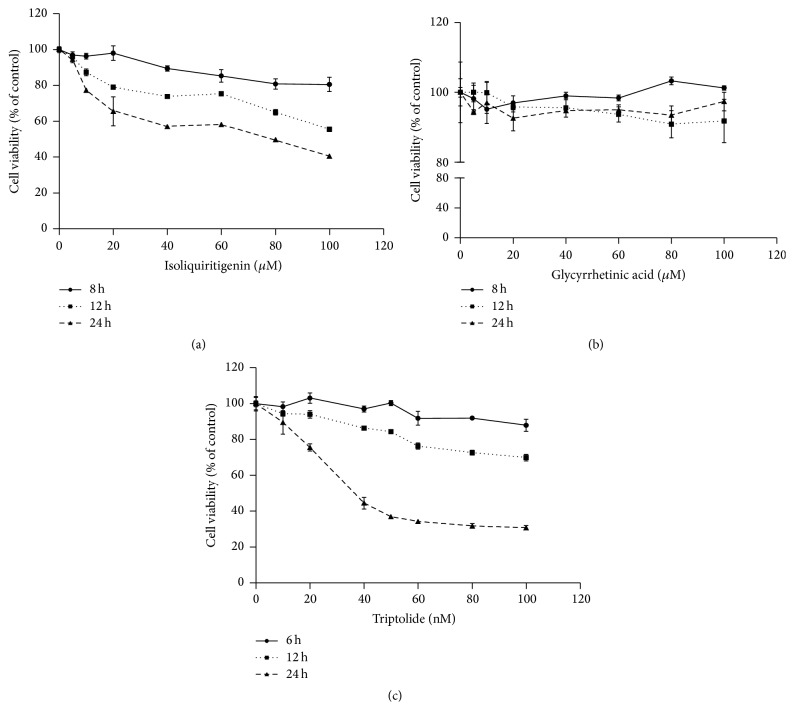
Cytotoxicity of ISL (a), GA (b), and TP (c) in HepG2 cells. Cells were exposed to various concentrations and time of tested drugs before being subjected to the MTT assay (*n* = 3).

HepG2 cells were treated with tested drugs and cell viability was measured using the MTT assay to evaluate the cytotoxicity effect. As shown in Figures 2(a) and 2(b), ISL decreased the cell viability in dose- and time-dependent manner while GA exhibited no obvious cytotoxicity after 12 h even 24 h of treatment. Based on this observation, the low dose (5, 10, and 20 *μ*M) of ISL and GA treatment for 12 h and 24 h, respectively, was chosen for the following research. After exposure to TP for desired time, the relative cell survival rate decreased dose-dependently (Figure 2(c)). Moreover, the IC_50_ (24 h) was 41.0 nM and a 24 h and the concentration of 50 nM were selected for the following experiment.

### 3.2. Oxidative Stress

**Figure 3:**
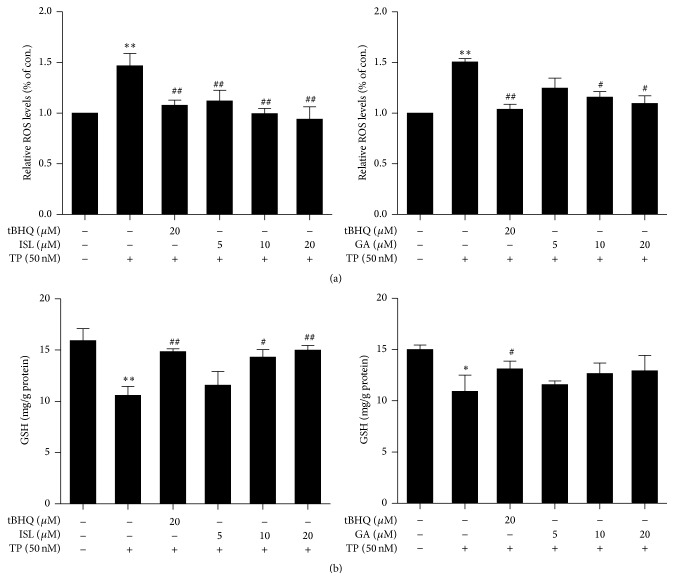
Cells were exposed to 50 nM TP for 24 h after incubation with various concentrations of ISL and GA for 12 h and 24 h, respectively. (a) The content of ROS was measured and normalized to control group. (b) The content of intracellular GSH was measured and normalized to protein content (*n* = 3). ^*∗*^ *p* < 0.05 versus control, ^*∗∗*^ *p* < 0.01 versus control; ^#^ *p* < 0.05 versus TP group, ^##^ *p* < 0.01 versus TP group. Control (Column 1): 0.1% DMSO.

The effects of ISL and GA combining with TP on ROS levels were assessed and tBHQ, a classical activator of Nrf2, was used as positive control. As shown in Figure 3(a), after 50 nM TP treatment for 24 h, the increasing levels of relative ROS were observed, which may lead to increasing probability of cellular damage caused by ROS. However, ISL and GA dose-dependently attenuated TP-induced ROS production compared with TP-treated alone.

### 3.3. Antioxidant Activity

The intracellular GSH content was detected to confirm the antioxidative activities of ISL and GA. TP significantly decreased intracellular GSH levels after 24 h of treatment as shown in Figure 3(b). Moreover, this decline was attenuated by administration of ISL and GA which both enhanced intracellular GSH content dose-dependently.

### 3.4. Effects of ISL and GA on the Protein Expression Nrf2 and Its Target Genes

**Figure 4:**
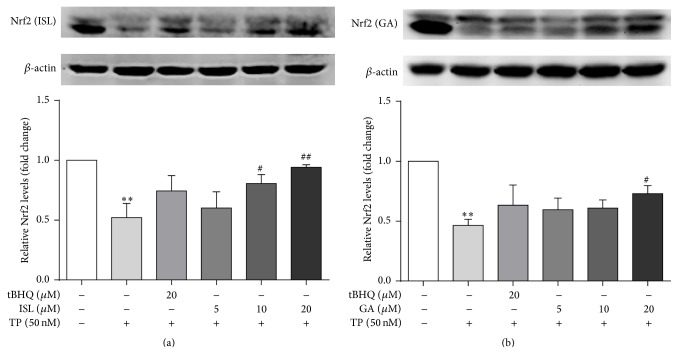
Cells were treated with different concentrations of ISL (a) and GA (b) for 12 h and 24 h, respectively, after which they were exposed to 50 nM TP for 24 h. The protein expression and gray value of Nrf2 was measured (*n* = 3). ^*∗∗*^ *p* < 0.01 versus control; ^#^ *p* < 0.05 versus TP group, ^##^ *p* < 0.01 versus TP group. Control (Column 1): 0.1% DMSO.

**Figure 5:**
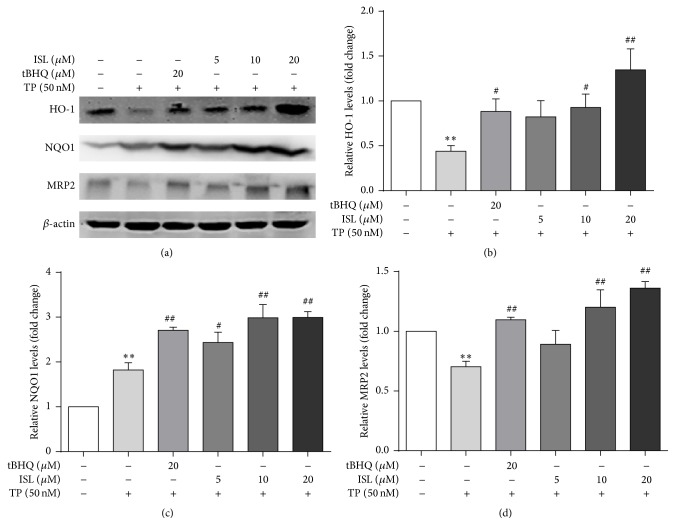
Cells were treated with different concentrations of ISL and tBHQ for 12 h, after which they were exposed to 50 nM TP for 24 h. The protein expressions (a) and gray value of HO-1 (b), NQO1 (c), and MRP2 (d) were measured (*n* = 3). ^*∗∗*^ *p* < 0.01 versus control; ^#^ *p* < 0.05 versus TP group, ^##^ *p* < 0.01 versus TP group. Control (Column 1): 0.1% DMSO.

**Figure 6:**
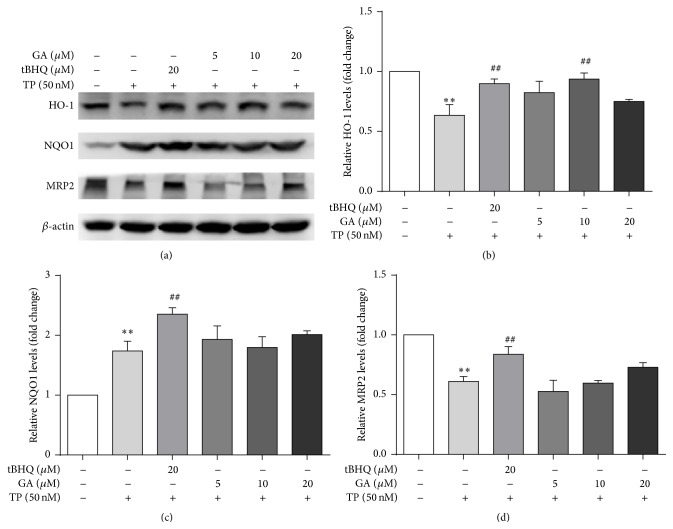
Cells were treated with different concentrations of GA and tBHQ for 24 h, after which they were exposed to 50 nM TP for 24 h. The protein expressions (a) and gray value of HO-1 (b), NQO1 (c), and MRP2 (d) were measured (*n* = 3). ^*∗∗*^ *p* < 0.01 versus control; ^##^ *p* < 0.01 versus TP group. Control (Column 1): 0.1% DMSO.

TP pronouncedly suppressed the expression of Nrf2 (Figure 4). In addition, the results indicated that the amount of protein expression of Nrf2 was increased dose-dependently in ISL and GA pretreatment groups compared with TP-treated group. Furthermore, the protein expression of the downstream genes including HO-1, NQO1, and MRP2 was measured (Figures 5 and 6). TP significantly decreased the protein abundance of HO-1 and MRP2 while the expression of NQO1 was significantly increased. Compared with TP-treated group, ISL and GA pretreatment group showed a marked increase in the expression of HO-1, NQO1, and MRP2 in a dose-dependent manner.

## 4. Discussion

TWHF is a well-known herbal medicine that is widely used to treat various diseases, including rheumatoid arthritis, nephritic syndrome, and lupus [1]. TP is a major active ingredient extracted and isolated from TWHF in 1972, and its pharmacological effects have been extensively investigated. But before this compound can reach its clinical potential, significant challenges remain to overcome, such as severe toxicities involving hepatotoxicity and poor aqueous solubility [13]. To enhance its efficacy and to decrease its toxicity, TWHF is frequently used in combination with other Chinese herbs, such as* Glycyrrhiza uralensis* Fisch. (Licorice) and* Astragalus membranaceus* (Fisch.) Bunge [14]. Licorice has also been a commonly used Traditional Chinese Medicine for many years. ISL and GA, two main active ingredients obtained from licorice, contain a Michael acceptor which is common to many ARE inducers [15]. The *α*,*β*-unsaturated carbonyl group has been shown to modify specific cysteine residues of Keap1, which confirm their activation effects on Nrf2 pathway structurally [16]. In addition, our laboratory previously found that ISL (among four compounds derived from licorice) was the most potent Nrf2 pathway inducer in HepG2 [12]. In the present study, we further conveyed that ISL and GA could activate the Nrf2 pathway, increase the protein expression of its target genes, and protect against TP-induced oxidative stress in HepG2 cells.

Activation of Nrf2 pathway plays pivotal role in preventing xenobiotic-related oxidative damage [17]. In current study, the protective role of Nrf2 was found to be attributed partly to its involvement in coordinated induction of phase II enzymes and phase III drug transporters. HO-1 catalyzes the first and rate-limiting step in the catabolism of the prooxidant heme to carbon monoxide, biliverdin, and free iron [18, 19]. It has been implicated to play a protective role in antioxidative activity following cellular injury and oxidative stress [20]. NQO1 is a cytosolic flavoprotein catalyzing the electroreductive metabolism and detoxification of endogenous and exogenous chemicals [21]. GCL, consisting of a modulatory (GCLM) and a catalytic (GCLC) subunit, is the rate-limiting enzyme for biosynthesis of GSH [22]. MRP2 participates in excretion of chemicals into bile, especially glutathione-, glucuronide-, and sulfate-conjugated metabolites [23]. All of these are important drug metabolism enzymes and transporters and played vital role in antioxidation and toxicant metabolism and excretion.

A lot of studies including ours had focused on the effect of licorice on Nrf2 pathway [12]. A study found that ISL had suppressive effect on lipopolysaccharide-induced inflammatory responses in murine macrophage which may be associated with inhibiting the Keap1, increasing Nrf2 translocation, and inducing the mRNA expression of UGT1A1, NQO1, and HO-1 [24]. On the other hand, GA delayed the progression of cisplatin-induced renal injury through upregulation of Nrf2 in the BALB/c mice [25]. Moreover, GA protected against CCL_4_-induced mice chronic liver fibrosis involving upregulation of Nrf2 [26]. Although numerous studies demonstrated that ISL and GA could activate Nrf2 pathway, the current study is the first to combine ISL and GA with TP to assess their effects on reducing TP-induced hepatotoxicity.

Nrf2 is antioxidant transcription factor leading to protection against oxidative stress by associating with AREs and inducting its target genes described above. The effect of TP to inhibit Nrf2 pathway has been demonstrated in heart tissues and Leydig cells [27, 28]. Consequently, the current study hypothesized that it is also the target of TP in HepG2 cells. It is worthy to notice that the protein expression of Nrf2, HO-1, and MRP2 decreased obviously suggesting that this event may result in lower induction of antioxidant defenses (Figure 5) [29]. In fact, the current results observed a decrease in the GSH content and protein expression of antioxidant enzyme HO-1 in HepG2 cells which is in accordance with a lower Nrf2 activation. However, the results are inconsistent with recent reports in which the expressions of Nrf2 and HO-1 in BALB/C mice were slightly higher than those of control [7]. The difference may be due to different treatment times and experimental subjects. Moreover, increasing protein expression of NQO1 was observed in TP-treated group, indicated that NQO1 may be regulated by other transcription factors. In addition, it is interesting to mention that the protein expressions of HO-1, NQO1, and MRP2 in high dose of ISL-pretreated group were slightly higher compared with tBHQ-treated group. It indicated that the regulation of HO-1, NQO1, and MRP2 expressions by ISL might through independently direct interaction with Nrf2 and other factors such as HIF-1 [30] be involved, but this remains to be elucidated.

There are a few limitations of our study due to the absent of in vivo evaluation since TP-provoked hepatotoxicity is better to be approached in animal model rather than in HepG2 cell model. Moreover, the nuclear accumulation of Nrf2 was not detected to confirm nuclear translocation of Nrf2. Therefore, further studies will be carried out focusing on animals even on Nrf2−/− animals and the nuclear translocation of Nrf2 to further demonstrate the Nrf2 activation mechanisms of ISL and GA.

## 5. Conclusion

Collectively, the current study demonstrated that ISL and GA effectively attenuated TP-induced oxidative stress and Nrf2 pathway was involved in the protection. To our knowledge, the present study is the first to show that ISL and GA activate Nrf2-associated HO-1, NQO1, and MRP2 in HepG2 cells and exert antioxidative defense against TP-induced hepatotoxicity. Therefore, the results provide new and meaningful insight into protecting against oxidative stress induced by TP. Moreover, this study implies that Nrf2 pathway may present a new biological target and that ISL and GA might be candidates for the prevention of drug-induced hepatotoxicity partly via Nrf2 pathway.

## References

[1] Li X.-J., Jiang Z.-Z., Zhang L.-Y. (2014). Triptolide: progress on research in pharmacodynamics and toxicology. *Journal of Ethnopharmacology*.

[2] Mei Z., Li X., Wu Q., Hu S., Yang X. (2005). The research on the anti-inflammatory activity and hepatotoxicity of triptolide-loaded solid lipid nanoparticle. *Pharmacological Research*.

[3] Ma Q. (2013). Role of Nrf2 in oxidative stress and toxicity. *Annual Review of Pharmacology and Toxicology*.

[4] Ma Q., He X. (2012). Molecular basis of electrophilic and oxidative defense: promises and perils of Nrf2. *Pharmacological Reviews*.

[5] Hwang G.-W. (2012). Role of intracellular defense factors against methylmercury toxicity. *Biological and Pharmaceutical Bulletin*.

[6] Shen G., Kong A.-N. (2009). Nrf2 plays an important role in coordinated regulation of Phase II drug metabolism enzymes and Phase III drug transporters. *Biopharmaceutics and Drug Disposition*.

[7] Li J., Shen F., Guan C. (2014). Activation of Nrf2 protects against triptolide-induced hepatotoxicity. *PLoS ONE*.

[8] Lu Y.-F., Liu J., Wu K. C., Qu Q., Fan G., Klaassen C. D. (2014). Overexpression of Nrf2 protects against microcystin-induced hepatotoxicity in mice. *PLoS ONE*.

[9] Kao T.-C., Wu C.-H., Yen G.-C. (2014). Bioactivity and potential health benefits of licorice. *Journal of Agricultural and Food Chemistry*.

[10] Li Y. S., Tong P. J., Ma H. Z., Dai Q. D., Guan T. R., Song X. W. (2006). Toxicity attenuation and efficacy potentiation effect of liquorice on treatment of rheumatoid arthritis with *Tripterygium wilfordii*. *Zhongguo Zhong Xi Yi Jie He Za Zhi*.

[11] Liu M.-X., Dong J., Yang Y.-J., Yang X.-L., Xu H.-B. (2005). Progress in research on triptolide. *Zhongguo Zhong Yao Za Zhi*.

[12] Gong H., Zhang B.-K., Yan M. (2015). A protective mechanism of licorice (*Glycyrrhiza uralensis*): isoliquiritigenin stimulates detoxification system via Nrf2 activation. *Journal of Ethnopharmacology*.

[13] Phillips P. A., Dudeja V., McCarroll J. A. (2007). Triptolide induces pancreatic cancer cell death via inhibition of heat shock protein 70. *Cancer Research*.

[14] Liu J. Q., Ren X. J., Shu J. C., Zhang R., Pan J. X. (2011). Advances in studies on compatibility of triptolide. *Jiangxi Journal of Traditional Chinese Medicine*.

[15] Cuendet M., Oteham C. P., Moon R. C., Pezzuto J. M. (2006). Quinone reductase induction as a biomarker for cancer chemoprevention. *Journal of Natural Products*.

[16] Luo Y., Eggler A. L., Liu D., Liu G., Mesecar A. D., van Breemen R. B. (2007). Sites of alkylation of human Keap1 by natural chemoprevention agents. *Journal of the American Society for Mass Spectrometry*.

[17] Lin M., Zhai X., Wang G. (2015). Salvianolic acid B protects against acetaminophen hepatotoxicity by inducing Nrf2 and phase II detoxification gene expression via activation of the PI3K and PKC signaling pathways. *Journal of Pharmacological Sciences*.

[18] Zhang J., Cao X., Ping S. (2015). Comparisons of ethanol extracts of Chinese propolis (poplar type) and poplar gums based on the antioxidant activities and molecular mechanism. *Evidence-Based Complementary and Alternative Medicine*.

[19] Hervera A., Leánez S., Motterlini R., Pol O. (2013). Treatment with carbon monoxide-releasing molecules and an HO-1 inducer enhances the effects and expression of *μ*-opioid receptors during neuropathic pain. *Anesthesiology*.

[20] Chang C.-F., Liu X.-M., Peyton K. J., Durante W. (2014). Heme oxygenase-1 counteracts contrast media-induced endothelial cell dysfunction. *Biochemical Pharmacology*.

[21] Lim J. H., Kim K.-M., Kim S. W., Hwang O., Choi H. J. (2008). Bromocriptine activates NQO1 via Nrf2-PI3K/Akt signaling: novel cytoprotective mechanism against oxidative damage. *Pharmacological Research*.

[22] Velmurugan G. V., Sundaresan N. R., Gupta M. P., White C. (2013). Defective Nrf2-dependent redox signalling contributes to microvascular dysfunction in type 2 diabetes. *Cardiovascular Research*.

[23] Vollrath V., Wielandt A. M., Iruretagoyena M., Chianale J. (2006). Role of Nrf2 in the regulation of the Mrp2 (ABCC2) gene. *Biochemical Journal*.

[24] Wang R., Zhang C. Y., Bai L. P. (2015). Flavonoids derived from liquorice suppress murine macrophage activation by up-regulating heme oxygenase-1 independent of Nrf2 activation. *International Immunopharmacology*.

[25] Wu C.-H., Chen A.-Z., Yen G.-C. (2015). Protective effects of glycyrrhizic acid and 18*β*-glycyrrhetinic acid against cisplatin-induced nephrotoxicity in BALB/c mice. *Journal of Agricultural and Food Chemistry*.

[26] Chen S., Zou L., Li L., Wu T. (2013). The protective effect of glycyrrhetinic acid on carbon tetrachloride-induced chronic liver fibrosis in mice via upregulation of Nrf2. *PLoS ONE*.

[27] Zhou J., Xi C., Wang W. (2014). Triptolide-induced oxidative stress involved with Nrf2 contribute to cardiomyocyte apoptosis through mitochondrial dependent pathways. *Toxicology Letters*.

[28] Hu J., Yu Q., Zhao F. (2015). Protection of Quercetin against triptolide-induced apoptosis by suppressing oxidative stress in rat leydig cells. *Chemico-Biological Interactions*.

[29] Carrasco-Pozo C., Castillo R. L., Beltrán C., Miranda A., Fuentes J., Gotteland M. (2016). Molecular mechanisms of gastrointestinal protection by quercetin against indomethacin-induced damage: role of NF-*κ*B and Nrf2. *The Journal of Nutritional Biochemistry*.

[30] Hjortsø M. D., Andersen M. H. (2014). The expression, function and targeting of haem oxygenase-1 in cancer. *Current Cancer Drug Targets*.

